# Cross-talk between ANGPTL4 gene SNP Rs1044250 and weight management is a risk factor of metabolic syndrome

**DOI:** 10.1186/s12967-021-02739-z

**Published:** 2021-02-16

**Authors:** Zhoujie Tong, Jie Peng, Hongtao Lan, Wenwen Sai, Yulin Li, Jiaying Xie, Yanmin Tan, Wei Zhang, Ming Zhong, Zhihao Wang

**Affiliations:** 1 The Key Laboratory of Cardiovascular Remodeling and Function Research, Chinese Ministry of Education, Chinese National Health Commission and Chinese Academy of Medical Sciences, The State and Shandong Province Joint Key Laboratory of Translational Cardiovascular Medicine, Department of Cardiology, Qilu Hospital, Cheeloo College of Medicine, Shandong University, Jinan, 250012 Shandong China; 2 Department of Geriatric Medicine, Qilu Hospital, Cheeloo College of Medicine, Shandong University; Shandong Key Laboratory of Cardiovascular Proteomics, Jinan, 250012 Shandong China

**Keywords:** Metabolic syndrome, Single nucleotide polymorphism, ANGPTL4, Weight management

## Abstract

**Background:**

The prevalence of metabolic syndrome (Mets) is closely related to an increased incidence of cardiovascular events. Angiopoietin-like protein 4 (ANGPTL4) is contributory to the regulation of lipid metabolism, herein, may provide a target for gene-aimed therapy of Mets. This observational case control study was designed to elucidate the relationship between *ANGPTL4* gene single nucleotide polymorphism (SNP) rs1044250 and the onset of Mets, and to explore the interaction between SNP rs1044250 and weight management on Mets.

**Methods:**

We have recruited 1018 Mets cases and 1029 controls in this study. The SNP rs1044250 was genotyped with blood samples, base-line information and Mets-related indicators were collected. A 5-year follow-up survey was carried out to track the lifestyle interventions and changes in Mets-related indicators.

**Results:**

*ANGPTL4* gene SNP rs1044250 is an independent risk factor for increased waist circumference (OR 1.618, 95% CI [1.119–2.340]; *p* = 0.011), elevated blood pressure (OR 1.323, 95% CI [1.002–1.747]; *p* = 0.048), and Mets (OR 1.875, 95% CI [1.363–2.580]; *p* < 0.001). The follow-up survey shows that rs1044250 CC genotype patients with weight gain have an increased number of Mets components (M [Q1, Q3]: CC 1 (0, 1), CT + TT 0 [− 1, 1]; *p* = 0.021); The interaction between SNP rs1044250 and weight management is a risk factor for increased systolic blood pressure (β = 0.075, *p* < 0.001) and increased diastolic blood pressure (β = 0.097, *p* < 0.001), the synergistic effect of weight management and SNP rs1044250 is negative (S < 1).

**Conclusion:**

*ANGPTL4* gene SNP rs1044250 is an independent risk factor for increased waist circumference and elevated blood pressure, therefore, for Mets. However, patients with wild type SNP 1044250 are more likely to have Mets when the body weight is increased, mainly due to elevated blood pressure.

## Background

With the global economy thriving, the Western diet and the sedentary living habits have been disseminated, and physical labor has been largely reduced. As a result, the prevalence of Mets has increased drastically in recent years [1]. The 2010–2012 Chinese National Nutrition and Health Survey suggested that the general prevalence of Mets has reached 24.2%, including 24.6% for men and 23.8% for women [2]. Mets is highly concerned as it doubles the risk of cardiovascular disease, while the all-cause mortality rate for Mets patients increases by 1.5 times [3]. Controlling the incidence of Mets and reducing complications is world-wide urgent. However, current Mets guidelines recommend treatments mainly based on lifestyle interventions, including smoking cessation, Mediterranean diet, 30–60 min of physical exercise per day, and a minimum of 5% weight-loss goal for obese patients [4]. Though the molecular-targeted drug therapy has been implemented for a variety of diseases, there is no specific drug for Mets treatment yet [4]. New options are urgently required for Mets, especially in the field of gene-targeted therapy.

Mets is a heterogenic and multifactorial diagnosis. The whole-gene linkage analysis is failed to identify loci that correspond to functional genes [5]. The Genome-Wide Association Study (GWAS) has inherent limitations on the minimum frequency of SNP, and the total effect of identified loci only explains a small proportion of Mets prevalence [5]. Under these circumstances, the SNP study is still the most important method for Mets gene research. It has been found that over 870 SNPs are associated with obesity [6], 477 SNPs with lipid metabolism [7], more than 200 SNPs with blood pressure [8], and around 250 SNPs with glucose tolerance [9]. However, most of these gene loci were related to single Mets components, among which the lipid metabolism-associated SNPs showed the strongest relevance to Mets [10]. Furthermore, current researches have been inadequate in gene-environmental interaction study. We expect to find a locus that participates in multiple components of Mets, and to carry out research on the interaction of gene polymorphism and environments.

As universally acknowledged, lipid metabolism, especially triglyceride (TG) metabolism plays a central role in Mets pathogenesis [11]. TG elevation is one of the components of Mets and a risk factor for abdominal obesity [12], and the synthesis of TG is associated with glucose metabolism through the tricarboxylic acid cycle [13]. Consequently, the gene locus featured by TG regulation is expected to become the target of Mets gene-directed therapy. Lipoprotein lipase (LPL) regulates TG hydrolysis in circulation and in adipose tissue. Angiopoietin-like protein 4 (ANGPTL4) is characterized by a reversible inhibitor of LPL [14]. It has been reported that ANGPTL4 is not only involved in the regulation of blood lipids [14], but also blood pressure [15, 16], glucose tolerance [17–20]. Therefore, the *ANGPTL4* gene is considered feasible for the gene polymorphism study for Mets.

**Fig. 1:**
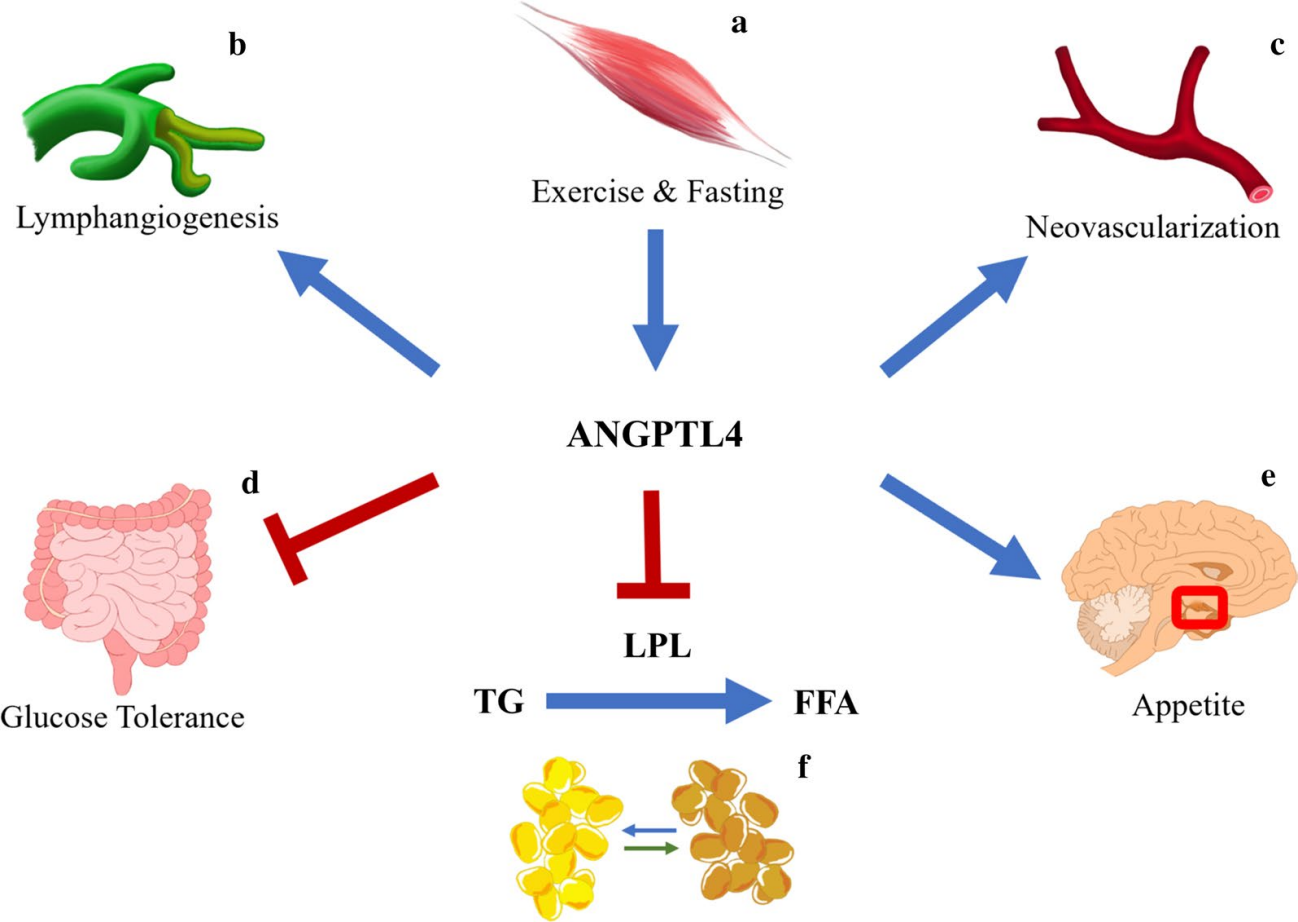
Roles of ANGPTL4. **a** Muscle-derived ANGPTL4 levels is increased during exercise or fasting; **b**, **c** ANGPTL4 involves in the process of neovascularization and lymphangiogenesis; **d** ANGPTL4 improves glucose tolerance through an intestinal microbiota dependent mechanism; **e** ANGPTL4 involves in the hypothalamic regulation of appetite, energy consumption and lipid deposition; **f** ANGPTL4 promotes the lipolysis of white adipose tissue by inhibiting lipoprotein lipase (LPL) activity, which mediate the decomposition of triglyceride (TG) to free fatty acid (FFA); in cold exposure, ANGPTL4 mediates the FFA transportation through white adipose tissue to brown adipose tissue

*ANGPTL4* gene which encodes the expression of ANGPTL4 locates on human chromosome 19. The N-terminal oligomerized ANGPTL4 plays an important role in the regulation of circulating TG levels by mediating LPL inhibition [21]. It has been reported that the *ANGPTL4* gene is related to obesity and weight management [22, 23]. ANGPTL4 involves in the regulation of body weight by white fat tissue (WAT) decomposition [24–28], and it participates in the up-regulation of circulating free fatty acids (FFA) during fasting and exercise [22, 29–31], plasma ANGPTL4 concentration is reported positively correlated with gestational weight gain [32]. Apart from the regulation of lipid metabolism and glucose tolerance, researchers have identified a peroxisome proliferator-activated receptors-regulated ANGPTL4 overexpression, which involves in the crosstalk between metabolism and cancer [33]. In addition, the C-terminus of ANGPTL4 interacts with extracellular matrix receptors through an N-linked glycan chain, which selectively prevents the activation of the cytokine cascade in endothelial cells, therefore, inhibits the process of neovascularization [15]. A figure has been constructed to capture the multifunctional characteristic of ANGPTL4 (Fig. 1), on account of which, we speculate that the *ANGPTL4* gene may become a potential target for Mets gene-directed study.

We selected a missense mutation rs1044250 (6959C > T, T266M) located on the highly conserved sequence encoding the C-terminus of *ANGPTL4*. The impact of SNP rs1044250 on the risks for Mets and its components were interpreted, afterward, the interactions between lifestyle interventions and SNP rs1044250 on Mets were investigated. We speculate that the study of ANGPTL4 gene polymorphism and lifestyle management is potentially helpful to elucidate the susceptibility of Mets, and may provide a feasible way for Mets intervention.

## Material and methods

### Subjects

Participants in this study were the Han population from Shandong Province, China, surveyed from January to December 2007. The sample size of this research was determined based on the prevalence of Mets and previously published frequency of SNP rs1044250, and a minimum sample size of 978 case–control pair was generated by QUANTO 1.2 (Gauderman J, Morrison J; University of Southern California). By numbering and random sampling, one rural county and one city region were randomly selected from Shandong province, two communities (around 500 to 1000 households each) from the chosen county and city were randomly selected respectively. Afterward, one individual from each household was randomly recruited and investigated. Eventually, a total of 1029 subjects in the control group and 1018 in the Mets group were recruited in this study. Of all the participants, 202 controls and 831 Mets cases participated in a 5-year follow-up survey. Blood samples were collected and Mets-related body indicators were measured by qualified investigators. Medical history and basic information of the study population were collected in the form of questionnaires. The diagnostic criteria of Mets referred to the jointly established diagnose of IDF and AHA/NHLBI in 2009. Participants were recruited into the control group and the Mets group according to the diagnostic criteria. Exclusion criteria included: secondary hypertension, severe heart failure, renal failure, heart valve disease, and malignant tumors. Patients with missing key information in questionnaire, invalid blood test indicators or genotype results were excluded.

### Basic data collection and laboratory examination

The questionnaire, physical examination and blood sample collection in the cross-section and follow-up surveys were all conducted using standard protocols. The questionnaire included detailed information on previously diagnosed diseases and medication history. Height, weight and waist circumference (WC) were measured in person by qualified surveyors. Omron HEM-7011 electronic sphygmomanometer (Omron, Dalian, China) was used to measure the blood pressure on the right arm after a 5-min rest in sitting position, three consecutive readings of each individual were recorded and the averages were calculated. Blood samples of participants were collected after overnight-fasting, Mets-related indicators were detected and the DNA extraction was performed in a standard laboratory. Beckman Coulter LX20 chemical analyzer (Beckman Coulter, Brea, CA) was used to determine the blood glucose. Genomic DNA was extracted using the blood DNA extraction kit D3133-03 (Magen, Guangzhou, China) according to the instructions. The genotype of rs1044250 was detected by the Sequenom MassArray genotyping system (Sequenom, San Diego, CA). Agarose gel electrophoresis was used to determine the quality of DNA extraction when the genotyping was failed.

### Mets interventions

Participants with increased body mass index (BMI), hypertension, diabetes, dyslipidemia, and other conditions related to Mets and cardiovascular risks were suggested to have weight management and lifestyle interventions, which include reducing dietary sugar and fat, having regular and moderate physical exercise, tobacco and alcohol cessation, visiting physician regularly. Prescription drugs including antihypertensives, hypoglycemics, and statins were also recommended as needed. The out-come of weight management was measured by changes in body weight (ΔWeight), and participants in the follow-up survey were subdivided into weight loss group (ΔWeight < 0) and weight gain (ΔWeight ≥ 0) group accordingly.

### Statistics

The χ^2^ goodness-of-fit test is used to test the Hardy–Weinberg equilibrium at rs1044250 locus. Continuous variables are presented in $\overline{{\text{x}}} \pm {\text{S}}$ and compared by independent-samples t test or one-way analysis of variances. Categorical variables are presented in proportions and compared by χ^2^ test, χ^2^ is calibrated by Bonferroni when the minimum sample size is lower than 5. Counting variables are presented in Median [Quartile1, Quartile3] (M [Q1, Q3]) and analyzed by Nonparametric test. Multi-factor logistic regression is used to analyze the risk factors for Mets and its components. The differentials in laboratory indicators after 5-year follow-up are calculated by subtracting the cross-section value from the follow-up value, differentials are recorded in "Δ". Multiple stepwise linear regression is used to analyze the effects of gene polymorphism on the number of Mets components and on changes of systolic blood pressure (SBP), diastolic blood pressure (DBP) and high-density lipid-c (HDL-c). Ordinal logistic regression is used to elucidate the impact of gene polymorphism on the number changes of Mets components. Crossover analysis is applied to analyze the interaction between two independent variables on Mets. All statistical analysis is operated using SPSS 26.0 (Chicago, Illinois SPSS). A two-tailed *p* value of less than 0.05 is considered to be statistically significant.

## Results

### Basic characteristics of the control and the Mets group

Participants in the control group (n = 1029) and the Mets group (n = 1018) are matched by gender and age, differences of the weight, WC, BMI, SBP, DBP, TG, total cholesterol (TC), low-density lipid-c (LDL-c), HDL-c, fasting plasma glucose (FPG) levels between the Control group and the Mets group are statistically significant (*p* < 0.001, Table 1). Frequencies of the C allele and the T allele on rs1044250 locus account for 92.1% and 7.9% respectively in the study population. Distribution of CC, CT, TT genotypes in the control group (χ^2^ = 0.02; *p* = 0.99), the Mets group (χ^2^ = 1.48; *p* = 0.48), and the study population (χ^2^ = 1.58; *p* = 0.45) are followed with Hardy–Weinberg genetic balance.

**Table 1:** Baseline characteristics of Control group and Mets group

	Controls (n = 1029)	Mets (n = 1018)	*p*
Male %	48.5	46	0.253
Age (years)	51.29 ± 9.78	51.64 ± 9.80	0.425
Weight (kg)	58.33 ± 8.30	72.60 ± 11.08	< 0.001
WC (cm)	75.68 ± 6.10	91.11 ± 8.46	< 0.001
BMI (kg/m^2^)	22.13 ± 3.46	27.32 ± 3.46	< 0.001
SBP (mmHg)	119.69 ± 9.90	148.50 ± 19.95	< 0.001
DBP (mmHg)	74.66 ± 6.44	90.53 ± 10.45	< 0.001
TC (mmol/L)	4.17 ± 0.53	4.68 ± 1.18	< 0.001
TG (mmol/L)	0.93 ± 0.31	2.32 ± 1.57	< 0.001
HDL-c (mmol/L)	1.59 ± 0.34	1.49 ± 0.52	< 0.001
LDL-c (mmol/L)	2.58 ± 0.52	3.19 ± 0.82	< 0.001
FPG (mmol/L)	4.59 ± 0.51	5.96 ± 2.11	< 0.001

Continuous variables are recorded by $\overline{x} \pm {\text{S}}$; categorical variables are recorded in proportions; Mets, metabolic syndrome; BMI, body mass index; WC, waist circumference; SBP, systolic blood pressure; DBP, diastolic blood pressure; TG, triglyceride; TC, total cholesterol; HDL-c, high-density lipoprotein-cholesterol; LDL-c, low-density lipoprotein-cholesterol; FPG, fasting plasma glucose

### Risks of the SNP rs1044250 on Mets and the components of Mets

The laboratory indicators among patients with CC, CT and TT genotypes at *ANGPTL4* gene rs1044250 locus are compared respectively, the result indicates that the baseline data among genotypes in the control group do not show difference (*p* > 0.05, Table 2), while in the Mets group, HDL-c is significantly higher in patients with TT genotype than patients with CC or CT genotypes (*p* < 0.05, Table 2).

**Table 2:** Baseline characteristics of various rs1044250 genotypes in the Control group and the Mets group

	Control (n = 1017)			Mets (n = 1018)		
	CC	CT	TT	CC	CT	TT
n	900	125	4	811	191	16
Male %	49.00%	44.80%	50.00%	47.70%	39.80%	31.30%
Age (years)	51.28 ± 9.82	51.13 ± 9.59	58.00 ± 2.45	51.30 ± 9.71	52.79 ± 9.96	54.94 ± 11.36
Weight (kg)	58.26 ± 8.29	58.90 ± 8.46	54.81 ± 5.01	72.97 ± 10.90	71.22 ± 11.55	69.94 ± 13.46
WC (cm)	75.61 ± 6.14	76.11 ± 5.92	77.63 ± 5.92	91.30 ± 8.54	90.23 ± 8.23	92.06 ± 6.74
BMI (kg/m2)	22.10 ± 2.57	22.36 ± 2.53	20.33 ± 1.17	27.40 ± 3.42	27.03 ± 3.57	26.87 ± 4.08
SBP (mmHg)	119.77 ± 10.11	119.21 ± 8.19	116.17 ± 13.11	148.45 ± 20.25	148.94 ± 19.15	145.67 ± 14.38
DBP (mmHg)	74.65 ± 6.49	74.77 ± 6.11	74.83 ± 6.66	90.74 ± 10.59	89.78 ± 10.81	88.67 ± 11.67
TC (mmol/L)	4.16 ± 0.54	4.22 ± 0.46	3.87 ± 0.71	4.66 ± 1.18	4.73 ± 1.15	5.09 ± 1.30
TG (mmol/L)	0.93 ± 0.31	0.97 ± 0.31	0.72 ± 0.20	2.35 ± 1.63	2.19 ± 1.27	2.32 ± 1.93
HDL (mmol/L)	1.59 ± 0.34	1.57 ± 0.31	1.54 ± 0.43	1.47 ± 0.51	1.51 ± 0.52	1.80 ± 0.86^a, b^
LDL (mmol/L)	2.57 ± 0.53	2.66 ± 0.53	2.33 ± 0.71	3.18 ± 0.83	3.20 ± 0.83	3.29 ± 0.75
FPG (mmol/L)	4.60 ± 0.52	4.55 ± 0.51	4.16 ± 0.38	5.95 ± 2.08	6.03 ± 2.24	5.68 ± 1.99

Continuous variables are recorded by $\overline{x} \pm {\text{S}}$; Categorical variables are recorded in proportions

Mets: metabolic syndrome; BMI: body mass index; WC: waist circumference; SBP: systolic blood pressure; DBP: diastolic blood pressure; TG: triglyceride; TC: total cholesterol; HDL-c: high-density lipoprotein-cholesterol; LDL-c: low-density lipoprotein-cholesterol; FPG: fasting plasma glucose

^a^Statistically significant compared with CC genotype in the Mets group

^b^Statistically significant compared with CT genotype in the Mets group

The frequencies of rs1044250 genotypes are significantly different between the control group and the Mets group (χ^2^ = 25.556; *p* < 0.001, Table 3), and the frequency of T alleles in the control group is significantly lower than that in the Mets group (χ^2^ = 25.991; *p* < 0.001, Table 3). The study population is subdivided into groups respectively according to the presence or absence of each of the five components of Mets. The frequencies of CC, CT, TT genotypes as well as the frequencies of C allele and T allele are significantly different between the normal WC group and the increased WC group, statistical significances were also found between the normal TG group and the elevated TG group, the normal blood pressure group and the elevated blood pressure group, the normal FPG group and the elevated FPG group (*p* < 0.05, Table 3).

**Table 3:** Distribution of SNP rs1044250 genotypes and alleles in Mets and Mets components

	n	Genotypes			χ^2^	*p*	Alleles		χ^2^	*p*
		CC	CT	TT			C	T		
Mets										
Control group	1029	900 (87.5%)	125 (12.1%)	4 (0.4%)	25.556	< 0.001	1925 (93.5%)	133 (6.5%)	25.991	< 0.001
Mets group	1018	811 (79.7%)	191 (18.8%)	16 (1.6%)			1847 (90.7%)	189 (9.3%)		
Component										
Normal BP	961	760 (86.9%)	111 (12.7%)	4 (0.5%)	13.68	0.001	1631 (93.2%)	119 (6.8%)	13.834	< 0.001
Elevated BP	1169	951 (81.1%)	205 (17.5%)	20 (1.0%)			2107 (89.1%)	237 (10.1%)		
Normal TG	1347	1164 (85.0%)	196 (14.3%)	10 (0.7%)	6.96	0.031	2524 (92.1%)	216 (7.9%)	6.888	0.009
Increased TG	688	547 (80.8%)	120 (17.7%)	10 (0.7%)			1214 (89.7%)	140 (10.3%)		
Normal WC	1318	1009 (86.8%)	149 (12.8%)	4 (0.3%)	26.308	< 0.001	2441 (92.6%)	195 (7.4%)	9.741	0.002
Increased WC	717	702 (79.3%)	167 (18.9%)	16 (1.8%)			1299 (90.6%)	135 (9.4%)		
Normal FPG	1721	1324 (84.8%)	222 (14.2%)	15 (1.0%)	7.504	0.023	2870 (91.9%)	252 (8.1%)	6.447	0.011
Increased FPG	314	387 (79.6%)	94 (19.3%)	5 (1.0%)			868 (89.3%)	104 (10.7%)		
Normal HDL	1811	1216 (83.5%)	225 (15.5%)	15 (1.0%)	0.15	0.928	2657 (91.2%)	255 (8.8%)	0.048	0.827
Decreased HDL	224	495 (83.8%)	91 (15.4%)	5 (0.8.%)			1081 (91.5%)	101 (8.5%)		

Mets, metabolic syndrome; BP, blood pressure; TG, triglyceride; HDL-c, high-density lipoprotein-cholesterol; WC, waist circumference; FPG, fasting plasma glucose

Multi-factor stepwise logistic regression analysis shows the SNP rs1044250 is an independent risk factor for metabolic syndrome (OR 1.875 [95% CI 1.363–2.580]; *p* < 0.001, Table 4). Accordingly, the five components of Mets are studied respectively, the SNP rs1044250 is an independent risk factor for increased WC (OR 1.618 [95% CI 1.119–2.340]; *p* = 0.011, Table 4) and increased blood pressure (OR 1.323 [95% CI 1.002–1.747]; *p* = 0.048, Table 4). In addition, patients with various rs1044250 genotypes are significantly different in the numbers of Mets components (M [Q1, Q3]: CC 2 [0, 3], CT 3 [0, 3], TT 3 [3, 3]; *p* = 0.001). Multivariate linear regression analysis confirms that the SNP rs1044250 (β = 0.044; *p* = 0.007), age (β = 0.068; *p* < 0.001), weight (β = 0.221; *p* < 0.001), BMI (β = 0.387; *p* < 0.001) and LDL-c (β = 0.200; *p* < 0.001) are independent risk factors for the increased number of Mets components carried by patients.

**Table 4:** Logistic regression analysis of Mets and Mets components

	Independent variables	β	p	OR	95% CI	
					Lower	Upper
Mets	Sex (male vs female)	− 0.812	< 0.001	0.444	0.313	0.630
	Age (years)	0.019	0.009	1.019	1.005	1.034
	Weight (kg)	0.081	< 0.001	1.085	1.055	1.115
	BMI (kg/m^2^)	0.351	< 0.001	1.420	1.308	1.541
	LDL-c (mmol/L)	1.210	< 0.001	3.355	2.699	4.171
	rs1044250	0.629	< 0.001	1.875	1.363	2.580
Increased WC	Sex (male vs female)	− 2.367	< 0.001	0.094	0.059	0.149
	Age (years)	0.033	< 0.001	1.034	1.015	1.053
	Weight (kg)	0.104	< 0.001	1.110	1.073	1.148
	BMI (kg/m^2^)	0.265	< 0.001	1.303	1.185	1.434
	TG (mmol/L)	0.328	< 0.001	1.388	1.225	1.573
	HDL-c (mmol/L)	− 0.372	0.029	0.689	0.493	0.963
	SBP (mmHg)	0.046	< 0.001	1.047	1.033	1.060
	DBP (mmHg)	0.068	< 0.001	1.070	1.047	1.094
	rs1044250	0.481	0.011	1.618	1.119	2.340
Elevated BP	Age (years)	0.035	< 0.001	1.036	1.024	1.048
	BMI (kg/m^2^)	0.327	< 0.001	1.387	1.338	1.438
	FPG (mmol/L)	0.308	< 0.001	1.361	1.237	1.497
	LDL-c (mmol/L)	0.535	< 0.001	1.708	1.439	2.028
	rs1044250	0.280	0.048	1.323	1.002	1.747
Increased TG	BMI (kg/m^2^)	0.095	< 0.001	1.099	1.051	1.150
	WC (cm)	0.065	< 0.001	1.067	1.048	1.086
	TC (mmol/L)	0.708	< 0.001	2.030	1.778	2.319
	FPG (mmol/L)	0.146	< 0.001	1.158	1.079	1.242
	DBP (mmHg)	0.049	< 0.001	1.050	1.038	1.062
	rs1044250	0.046	0.752	1.047	0.786	1.395
Decreased HDL-c	Sex (male vs female)	0.491	< 0.001	1.634	1.332	2.004
	WC (cm)	0.028	< 0.001	1.028	1.016	1.040
	DBP (mmHg)	0.022	< 0.001	1.022	1.012	1.032
	rs1044250	− 0.078	0.532	0.925	0.723	1.182
Increased FPG	WC (cm)	0.046	< 0.001	1.048	1.034	1.062
	TC (mmol/L)	0.647	< 0.001	1.911	1.601	2.281
	TG (mmol/L)	0.140	0.002	1.151	1.054	1.256
	HDL-c (mmol/L)	− 0.447	0.016	0.640	0.444	0.921
	SBP (mmHg)	0.013	0.001	1.013	1.005	1.021
	DBP (mmHg)	0.018	0.011	1.018	1.004	1.032
	rs1044250	0.126	0.361	1.135	0.866	1.487

rs1044250, (CC = 0, TC = 1, TT = 2); OR, odds ratio; 95% CI, 95% confidence interval

Mets: metabolic syndrome; BMI: body mass index; SBP: systolic blood pressure; DBP: diastolic blood pressure; TG: triglyceride; TC: total cholesterol; HDL-c: high-density lipoprotein-cholesterol; LDL-c: low-density lipoprotein-cholesterol; WC: waist circumference; FPG: fasting plasma glucose

### Baseline characteristic changes of the study population

Compared with the control group, the weight, WC, BMI, SBP, DBP, TG, TC, LDL-c and FPG of the Mets group are significantly reduced in 5 years (Table 5). In the Mets group, HDL-c level is significantly elevated in patients with TT genotype than that in CC genotype or CT genotype (*p* < 0.05, Table 5). Linear regression analysis shows that the SNP rs1044250 (β = − 0.065; *p* = 0.017), Sex (β =  − 0.069; *p* = 0.012) and TG (β = − 0. 611; *p* < 0.001) are independent risk factors for HDL-c reduction in patients with Mets in the 5-year follow-up survey.

**Table 5:** Baseline characteristics changes in 5-year follow-up survey of various rs1044250 genotypes in the Control group and the Mets group

	Control			Mets			
	CC (n = 174)	CT + TT (n = 28)	Total (n = 202)	CC (n = 667)	CT (n = 151)	TT (n = 13)	Total (n = 202)
Sex (male%)	47.7%	57.1%	49.0%	47.5%	39.7%	30.8%	45.9%
Age (years)	51.64 ± 9.30	51.07 ± 10.51	51.56 ± 9.45	51.42 ± 9.68	52.47 ± 10.20	54.00 ± 10.57	51.65 ± 9.79
ΔWeight (kg)	− 0.31 ± 1.64	− 0.52 ± 1.66	− 0.37 ± 1.64	− 2.11 ± 3.11	− 1.98 ± 2.78	− 1.90 ± 1.50	− 2.08 ± 3.03^c^
ΔBMI (kg/m^2^)	0.09 ± 0.86	− 0.02 ± 0.65	0.07 ± 0.84	− 0.55 ± 1.08	− 0.51 ± 1.00	− 0.46 ± 0.63	− 0.54 ± 1.06^c^
ΔWC (cm)	− 0.54 ± 3.02	− 0.98 ± 2.24	− 0.60 ± 2.92	− 4.37 ± 3.36	− 3.91 ± 3.08	− 4.27 ± 3.45	− 4.28 ± 3.31^c^
ΔSBP (mmHg)	1.07 ± 6.79	0.17 ± 5.53	0.95 ± 6.62	− 7.16 ± 13.39	− 6.90 ± 11.75	− 4.13 ± 8.09	− 7.06 ± 13.04^c^
ΔDBP (mmHg)	− 0.27 ± 2.53	0.38 ± 1.59	− 0.18 ± 2.43	− 5.46 ± 6.85	− 5.17 ± 6.69	− 4.97 ± 6.96	− 5.40 ± 6.82^c^
ΔTG (mmol/L)	0.32 ± 0.50	0.23 ± 0.58	0.31 ± 0.51	− 0.84 ± 1.81	− 0.70 ± 1.43	− 1.02 ± 1.90	− 0.82 ± 1.75^c^
ΔTC (mmol/L)	0.13 ± 1.08	− 0.08 ± 1.15	0.10 ± 1.09	− 0.33 ± 1.31	− 0.21 ± 1.33	− 0.75 ± 1.61	− 0.32 ± 1.32^c^
ΔHDL-c (mmol/L)	− 0.24 ± 0.48	− 0.09 ± 0.36	− 0.22 ± 0.46	− 0.25 ± 0.66	− 0.32 ± 0.64	− 0.83 ± 1.12^a, b^	− 0.27 ± 0.67
ΔFPG (mmol/L)	0.43 ± 1.42	0.59 ± 1.34	0.45 ± 1.41	− 0.12 ± 2.43	− 0.38 ± 2.53	− 0.10 ± 1.82	− 0.17 ± 2.44^c^

Continuous variables are recorded by $\stackrel{-}{x}\pm S$; Categorical variables are recorded in proportions

Mets: metabolic syndrome; BMI: body mass index; WC: waist circumference; SBP: systolic blood pressure; DBP: diastolic blood pressure; TG: triglyceride; TC: total cholesterol; HDL-c: high-density lipoprotein-cholesterol; FPG: fasting plasma glucose

^a^Statistically significant compared with CC genotype in the Mets group

^b^Statistically significant compared with CT genotype in the Mets group

^c^Statistically significant compared with the Mets group; Δ, follow-up values minus baseline values

### Changes in the number of Mets components

The study population is subdivided into Weight loss group and Weight gain group, the distribution of rs1044250 genotypes (fisher = 1.162; *p* = 0.532) and T alleles (χ^2^ = 0.878; *p* = 0.349) in these groups do not show a significant difference. Among patients with CC, CT and TT genotypes at rs1044250 locus, changes of the number of Mets components do not show any difference (M [Q1, Q3]: CC 0 [− 1, 1], CT 0 [− 1, 1], TT 0 [− 1, 1]; *p* = 0.529, Table 6). However, subgroup analysis based on weight management indicates that the number changes of Mets components between CC genotype and CT or TT genotype in the weight gain group are significantly different (M [Q1, Q3]: CC 1 (0, 1), CT + TT 0 [− 1, 1]; *p* = 0.021, Table 6), while there is no difference showed in the weight loss group (M [Q1, Q3]: CC 0 [− 1, 1], CT + TT − 0 [− 1, 1], *p* = 0.732, Table 6). Indeed, under an ordinal regression model, the SNP rs1044250 (β = − 0.703; *p* = 0.024), TG (β = − 0.337; *p* < 0.001) and FPG (β = − 0.242; *p* = 0.003) are independent protective factors for the number of Mets components when the body weight is increased, which suggests that people with CC genotype are more likely to catch-up with the number of Mets component in the 5-year survey.

**Table 6:** The number changes of Mets components carried by various subgroups

	rs1044250	n	M [Q1, Q3]	MR	*p*
Follow-up	CC	841	0 [− 1, 1]	518.98	0.529
	CT	178	0 [− 1, 1]	502.31	
	TT	14	0 [− 1, 1]	585.14	
Weight loss	CC	709	0 [− 1, 1]	432.17	0.732
	TT + CT	157	0 [− 1, 1]	439.49	
Weigh gain	CC	132	1 (0, 1)	88.28	0.021
	TT + CT	35	0 [− 1, 1]	67.86	
Weight loss	CC	709	0 [− 1, 1]	397.24	< 0.001
Weigh gain	CC	132	1 [0, 1)	548.63	
Weight loss	TT + CT	157	0 [− 1, 1]	94.67	0.317
Weigh gain	TT + CT	35	0 [− 1, 1]	104.73	

M [Q1, Q3], Median [Quartile1, Quartile3]; MR, mean ranks

### Interaction of SNP rs1044250 and weight management

In the weight loss group, the HDL-c level is significantly decreased in patients with TT genotype at rs1044250 locus compared with CC (*p* = 0.002, Table 7) and CT (*p* = 0.008, Table 7) genotype. In the weight gain group, the SBP (*p* = 0.002, Table 7) and DBP (Table 7; *p* = 0.004) of CC genotype are significantly higher than that of CT genotype. Accordingly, the interaction of SNP rs1044250 and weight management on SBP (F = 3.291; *p* = 0.038, Table 7), DBP (F = 3.026; *p* = 0.049, Table 7) and HDL-c (F = 6.269; *p* = 0.002, Table 7) are statistically significant.

**Table 7:** Factorial analysis SNP rs1044250 and weight management on clinical characteristic changes

	Weight loss			Weight gain			*p*		
	CC (n = 709)	CT (n = 146)	TT (n = 11)	CC (n = 132)	CT (n = 32)	TT (n = 3)	rs1044250	ΔWeight	rs1044250 *ΔWeight
ΔWeight (kg)	− 2.23 ± 2.81	− 2.27 ± 2.68	− 2.25 ± 1.35	0.94 ± 2.24	0.53 ± 0.81	0.67 ± 1.15			
ΔBMI (kg/m^2^)	− 0.61 ± 0.94	− 0.61 ± 0.97	− 0.62 ± 0.51	0.61 ± 1.16	0.36 ± 0.38	0.55 ± 0.54			
ΔWC (cm)	− 4.46 ± 3.11	− 4.26 ± 2.76	− 5.45 ± 2.05	1.10 ± 2.33	0.20 ± 2.11	0.67 ± 2.75			
ΔSBP (mmHg)	− 6.48 ± 13.12	− 5.79 ± 10.81	− 4.55 ± 8.55	0.04 ± 8.77	− 6.12 ± 13.51^c^	0.33 ± 5.86	0.113	0.191	0.038
ΔDBP (mmHg)	− 5.06 ± 6.70	− 4.51 ± 6.40	− 5.97 ± 7.13	− 0.78 ± 4.14	− 3.51 ± 6.99 ^c^	0.56 ± 0.84	0.285	0.007	0.049
ΔTG (mmol/L)	− 0.69 ± 1.69	− 0.58 ± 1.39	− 1.33 ± 1.91	− 0.14 ± 1.64	− 0.48 ± 1.33	0.79 ± 0.31			
ΔTC (mmol/L)	− 0.30 ± 1.28	− 0.21 ± 1.36	− 0.80 ± 1.74	0.07 ± 1.22	− 0.17 ± 0.89	0.59 ± 1.97			
ΔHDL-c (mmol/L)	− 0.25 ± 0.64	− 0.31 ± 0.59	− 1.07 ± 0.97^a, b^	− 0.22 ± 0.53	− 0.19 ± 0.67	0.41 ± 0.83	0.889	< 0.001	0.002
ΔFPG (mmol/L)	− 0.04 ± 2.32	− 0.24 ± 2.47	− 0.12 ± 1.98	0.16 ± 1.96	− 0.21 ± 2.16	0.34 ± 0.75			

Continuous variables are recorded by $\stackrel{-}{x}\pm S$

Δ: follow-up values minus baseline values; BMI: body mass index; WC: waist circumference; SBP: systolic blood pressure; DBP: diastolic blood pressure; TG: triglyceride; TC: total cholesterol; HDL-c: high-density lipoprotein-cholesterol; FPG: fasting plasma glucose

^a^Statistically significant compared with CC genotype in the Weight loss group; ^b^Statistically significant compared with CT genotype in the Weight loss group; ^c^Statistically significant compared with CC genotype in the Weight gain group

The independent variable ΔWeight*rs1044250 is included in the multi-factor stepwise linear regression equations, of which the dependent variable is ΔSBP and ΔDBP respectively. Thereafter, the interaction of SNP rs1044250 and weight management is an independent risk factor for elevated SBP (β = 0.075; *p* < 0.001, Table 8) and elevated DBP (β = 0.097; *p* < 0.001, Table 8). The independent variable ΔWeight*rs1044250 is excluded from the linear regression equations of ΔHDL-c (β = 0.004, *p* = 0.851, Table 8).

**Table 8:** Linear regressions analysis of ΔSBP, ΔDBP and ΔHDL-c

	β	p	R^2^
ΔSBP (mmHg)			
Antihypertensive	− 0.215	< 0.001	0.719
Antidiabetic	0.058	0.001	
WC (cm)	0.135	< 0.001	
BMI (kg/m^2^)	0.052	0.032	
TG (mmol/L)	0.047	0.006	
SBP (mmHg)	− 0.835	< 0.001	
DBP (mmHg)	0.112	< 0.001	
ΔWeight*rs1044250	0.075	< 0.001	
ΔDBP (mmHg)			
Antihypertensive	− 0.373	< 0.001	0.696
Antidiabetic	0.048	0.007	
WC (cm)	0.148	< 0.001	
HDL-c (mmol/L)	− 0.035	0.045	
DBP (mmHg)	− 0.621	< 0.001	
ΔWeight*rs1044250	0.097	< 0.001	
ΔHDL-c (mmol/L)			
Antidiabetic	− 0.060	0.003	0.580
WC (cm)	− 0.072	0.003	
TG (mmol/L)	− 0.052	0.013	
HDL-c (mmol/L)	− 0.741	< 0.001	
ΔWeight*rs1044250	0.004	0.851	

Δ, follow-up values minus baseline values

BMI: body mass index; SBP: systolic blood pressure; DBP: diastolic blood pressure; TG: triglyceride; HDL-c: high-density lipoprotein-cholesterol; WC: waist circumference; FPG: fasting plasma glucose; rs1044250, (CC = 0, TC = 1, TT = 2)

A crossover analysis is conducted to study the interaction of ANGPTL4 gene SNP rs1044250 and weight management on ΔSBP, ΔDBP, ΔHDL-c under linear regression model. In the linear regression model with ΔSBP as the dependent variable, the independent variables SNP rs1044250 (β = 0.193; *p* < 0.001, Table 9) and rs1044250*Weight Management (β = − 0.093; *p* = 0.013, Table 9) are included in the regression equation, and the interaction between SNP rs1044250 and weight management on ΔSBP is negative (synergy index = 0.558); in the linear regression model with ΔDBP as the dependent variable, the SNP rs1044250 (β = 0.241; *p* < 0.001, Table 9) and rs1044250* Weight Management (β = − 0.078; *p* = 0.035, Table 9) are included in the regression equation, and the synergistic effect between SNP rs1044250 and weight management are negative (synergy index = 0.696).

**Table 9:** Crossover analysis of SNP rs1044250 and WM on ΔSBP, ΔDBP and ΔHDL-c

	β	OR	*p*	S
ΔSBP				
WM	0.024	1.024	0.477	0.558
Dom	0.193	1.212	< 0.001	
Dom*WM	− 0.093	0.911	0.013	
ΔDBP				
WM	0.027	1.027	0.429	0.696
Dom	0.241	1.273	< 0.001	
Dom*WM	− 0.078	0.925	0.035	
ΔHDL-c				
WM	− 0.066	0.936	0.053	0.106^a^
Dom	0.017	1.017	0.620	
Dom*WM	0.054	1.056	0.154	

SBP: systolic blood pressure; DBP: diastolic blood pressure; TG: triglyceride; HDL-c: high-density lipoprotein-cholesterol; WM: Weight loss = 0, Weight gain = 1; Dom, CC = 0, CT + TT = 1, OR: odds ratio; S: synergy index; S = [EXP(β_WM_ + β_Dom_ + β_Dom*WM_) − 1]/[EXP(β_WM_) + EXP(β_Dom_)—2]

^a^The denominator is calibrated from negative to positive

## Discussion

This is the first study that comprehensively identifies the *ANGPTL4* gene SNP rs1044250 as an independent risk factor for Mets by increasing the WC and blood pressure. The number of Mets components in CC genotype individuals increases when body weight raised. Consistently the SNP rs1044250 and weight management are negatively correlated on the interaction with blood pressure.

In 2007, a GWAS study found that *FTO* polymorphism was associated with weight gain and increased BMI [34]. It was the first GWAS study which ushered in the era of gene SNP research on Mets. Since then, the effects of gene polymorphisms have been related to obesity [6], lipid metabolism [7], glucose tolerance and hypertension [8, 9]. Because of the heterogeneity and multifactorial nature of Mets, current SNP studies have been limited on single Mets components, the effects of multifunctional gene loci on Mets are seldom reported. Under this circumstance, various studies have suggested that ANGPTL4 is involved in the regulation of multiple components of Mets, including lipid metabolism [14], obesity [24], blood pressure and glucose tolerance [15–17]. Regarding the multifunctional feature, the major role of ANGPTL4 is regulating the TG content in circulation and maintaining the balance of adipopexis in WAT [35]. The N-terminal oligomerized ANGPTL4 in circulation inhibits LPL activity, therefore, increases the circulating TG level [2], while the SNP rs1044250 mainly alters the activity of ANGPTL4 C-terminus [36]. Previous study has reported that the SNP rs1044250 only accounts for 0.8% of patients with decreased serum TG, and the TG reducing effect of SNP rs1044250 will not be significant after the impact of ANGPTL4 N-terminus related SNP rs110843064 (E40K) is excluded, whereas E40K is positively related to an increased overall coronary heart disease risk [2, 37]. Accordingly, our result suggests that the rs1044250 polymorphism does not have independent interaction with circulating TG, but Mets.

This study proves that the SNP rs1044250 is an independent risk factor for increased WC. It has been reported previously that overexpression of purified ANGPTL4 C-terminus in mouse accelerates the decomposition of WAT lipid, suggesting a lipolytic activity of ANGPTL4 C-terminal domain in fat cells independent from LPL [38]. Therefore, the SNP rs1044250 is likely to induce an increase in WC by lowering the level of WAT lipolysis. In addition, the WC and waist-to-hip ratio is increased in adipocyte ANGPTL4 knockout mice [22]. Another research has found that the ANGPTL4 knockout mice fed with high-fat diet show granuloma lesions in the intestine and WAT, as well as lymphangitis and mesenteric lymphadenitis [39, 40], which suggests that ANGPTL4 is essential to the lymphatic drainage of lipids from WAT to the liver. A study has also reported that ANGPTL4 reduces appetite by inhibiting hypothalamic adenosine monophosphate-activated protein kinase (AMPK) activity, accordingly, the ANGPTL4 knockout mice show increased appetite after fasting [41]. Regarding features of ANGPTL4 discussed above, we speculate that SNP rs1044250 causes abdominal fat accumulation and increased WC by promoting the WAT lipid recruitment, hindering lipolysis, destroying the integrity of WAT-hepatic lymphoid tissue as well as increasing appetite.

The ANGPTL4 gene SNP rs1044250 is an independent risk factor for elevated blood pressure. It has been reported that the C-terminal domain of ANGPTL4 protein inhibits vascular epithelial growth factor (VEGF) and basic fibroblast growth factor (bFGF)-mediated angiogenesis [15], and SNP rs1044250 is capable of altering the activity of ANGPTL4 C-terminus, therefore, the SNP rs1044250 may lead to a decreased angiogenesis and dysfunctional endothelial repairment. ANGPTL4 gene knockout mice are prone to have coronary arteritis and mesenteric vasculitis when fed with a high-fat diet [39, 42, 43]. The dysfunction of endothelial repairment can increase vascular resistance and lead to an increased blood pressure in both direct and indirect manners. Consistently, circulating ANGPTL4 protein levels are significantly up-regulated in patients with hypertension [16, 24]. Based on the risk of the SNP rs1044250 on WC and blood pressure, the ANGPTL4 gene polymorphism is considered a risk factor for Mets. A step forward, we attempt to cast light on the interaction between ANGPTL4 polymorphisms and lifestyle interventions, and to find a feasible way for rs1044250-targeted Mets therapy.

At present, the management of Mets is mainly based on lifestyle interventions. The body weight reflects a time superposition effect of lifestyle. Therefore, weight management has become a recommended indicator of lifestyle interventions to Mets. In 2017, the international panel recommended a minimum 5% weight loss target for obese patients [4]. Our result shows that patients with rs1044250 CC genotype are more likely to have an increased number of Mets components as body weight raised. Elevated blood pressure is considered the major cause of increased number of Mets components. Accordingly, the synergistic effect of weight management and SNP rs1044250 on blood pressure is negative, in other words, the superimposed effect of these two independent variables is less than the sum of their effects alone. Studies have shown that muscle-derived ANGPTL4 levels are increased during exercise or fasting [31, 44], and that WAT lipolysis is positively correlated to the circulating ANGPTL4 levels [22]. We speculate the lack of exercise leads to a decrease in myogenic ANGPTL4, which in turn results in the accumulation of WAT and weight gain. Therefore, the transient decrease of ANGPTL4 level in circulation during weight gain is negatively correlated to the risk of SNP rs1044250 on blood pressure. Under this circumstance, the blood pressure of rs1044250 wild-type patients shows a catch-up effect, which may relate to a fasting involved metabolism disorder.

Participants with Mets were suggested to have lifestyle interventions and take prescription drugs. As a result, the Mets group showed improvement in various physical and laboratory indicators in the follow-up survey. Participants with TT genotype have elevated HDL-c, which was significantly decreased among the weight loss group in the follow-up survey. ANGPTL4 is characterized by its lipolytic effect and HDL-c is negatively correlated to TG [26]. However, previous research has reported that elevated HDL-c is associated with ANGPTL4 overexpression [22], which is considered discrepant to the role of ANGPTL4. In this research, we have demonstrated a catch-up of HDL-c among participants with CC and CT genotype, in another word, weight management overtakes the negative correlation of r1044250 to HDL-c, unfortunately, the cross-over assay was failed to provide more evidence.

This study elaborated on the interaction between the ANGPTL4 gene SNP rs1044250 and weight management on Mets. Future studies need to clarify the effect of SNP rs1044250 on the structure of ANGPTL4 C-terminus, and to detect the interaction between SNP rs1044250 and neovascularization. The effect of SNP rs1044250 on circulating lipid metabolism under fasting and exercise conditions also requires experimental verification.

## Conclusion

We have found that the ANGPTL4 gene SNP rs1044250 increases the incidence of Mets in the Shandong Han population by increasing blood pressure and WC. The number of Mets components in patients with CC genotype at rs1044250 locus shows a catch-up effect when the body weight is increased, while weight-loss could significantly inhibit the increase of SBP and DBP caused by rs1044250 polymorphism.

## Data Availability

All data generated or analyzed during this study are included in this published article, the exclusive access to the original data is preserved by Zhihao Wang, and could be viewed under reasonable request.

## Abbreviations

Mets: Metabolic syndrome
SNP: Single nucleotide polymorphism
ANGPTL4: Angiopoietin-like protein 4
WAT: White adipose tissue
LPL: Lipoprotein lipase
GWAS: Genome-wide association study
BMI: Body mass index
SBP: Systolic blood pressure
DBP: Diastolic blood pressure
TG: Triglyceride
TC: Total cholesterol
HDL-c: High-density lipoprotein-cholesterol
LDL-c: Low-density lipoprotein-cholesterol
WC: Waist circumference
FPG: Fasting plasma glucose
*FTO*: Fat mass and obesity-associated protein
VEGF: Vascular endothelial growth factor
bFGF: Basic fibroblast growth factor

## References

[1] Saklayen MG (2018). The global epidemic of the metabolic syndrome. Curr Hypertens Rep.

[2] Talmud PJ, Smart M, Presswood E, Cooper JA, Nicaud V, Drenos F, Palmen J, Marmot MG, Boekholdt SM, Wareham NJ (2008). ANGPTL4 E40K and T266M: effects on plasma triglyceride and HDL levels, postprandial responses, and CHD risk. Arterioscler Thromb Vasc Biol.

[3] Mottillo S, Filion KB, Genest J, Joseph L, Pilote L, Poirier P, Rinfret S, Schiffrin EL, Eisenberg MJ (2010). The metabolic syndrome and cardiovascular risk a systematic review and meta-analysis. J Am Coll Cardiol.

[4] Pérez-Martínez P, Mikhailidis DP, Athyros VG, Bullo M, Couture P, Covas MI, de Koning L, Delgado-Lista J, Díaz-López A, Drevon CA (2017). Lifestyle recommendations for the prevention and management of metabolic syndrome: an international panel recommendation. Nutr Rev.

[5] Abou Ziki MD, Mani A (2016). Metabolic syndrome: genetic insights into disease pathogenesis. Curr Opin Lipidol.

[6] Rohde K, Keller M, la Cour PL, Blüher M, Kovacs P, Böttcher Y (2019). Genetics and epigenetics in obesity. Metabolism.

[7] Hoffmann TJ, Theusch E, Haldar T, Ranatunga DK, Jorgenson E, Medina MW, Kvale MN, Kwok PY, Schaefer C, Krauss RM (2018). A large electronic-health-record-based genome-wide study of serum lipids. Nat Genet.

[8] Wang Y, Wang JG (2019). Genome-Wide Association Studies of Hypertension and Several Other Cardiovascular Diseases. Pulse (Basel).

[9] Langenberg C, Lotta LA (2018). Genomic insights into the causes of type 2 diabetes. Lancet.

[10] Kristiansson K, Perola M, Tikkanen E, Kettunen J, Surakka I, Havulinna AS, Stancáková A, Barnes C, Widen E, Kajantie E (2012). Genome-wide screen for metabolic syndrome susceptibility Loci reveals strong lipid gene contribution but no evidence for common genetic basis for clustering of metabolic syndrome traits. Circ Cardiovasc Genet.

[11] Iqbal J, Al Qarni A, Hawwari A, Alghanem AF, Ahmed G (2018). Metabolic syndrome, dyslipidemia and regulation of lipoprotein metabolism. Curr Diabetes Rev.

[12] Jung MK, Yoo EG (2018). Hypertriglyceridemia in obese children and adolescents. J Obes Metab Syndr.

[13] Carreau AM, Jin ES, Garcia-Reyes Y, Rahat H, Nadeau KJ, Malloy CR, Cree-Green M (2019). A simple method to monitor hepatic gluconeogenesis and triglyceride synthesis following oral sugar tolerance test in obese adolescents. Am J Physiol Regul Integr Comp Physiol.

[14] Grootaert C, Van de Wiele T, Verstraete W, Bracke M, Vanhoecke B (2012). Angiopoietin-like protein 4: health effects, modulating agents and structure-function relationships. Expert Rev Proteomics.

[15] Yang YH, Wang Y, Lam KS, Yau MH, Cheng KK, Zhang J, Zhu W, Wu D, Xu A (2008). Suppression of the Raf/MEK/ERK signaling cascade and inhibition of angiogenesis by the carboxyl terminus of angiopoietin-like protein 4. Arterioscler Thromb Vasc Biol.

[16] Abu-Farha M, Cherian P, Qaddoumi MG, AlKhairi I, Sriraman D, Alanbaei M, Abubaker J (2018). Increased plasma and adipose tissue levels of ANGPTL8/Betatrophin and ANGPTL4 in people with hypertension. Lipids Health Dis.

[17] Morris A (2018). Obesity: ANGPTL4-the link binding obesity and glucose intolerance. Nat Rev Endocrinol.

[18] Gusarova V, O'Dushlaine C, Teslovich TM, Benotti PN, Mirshahi T, Gottesman O, Van Hout CV, Murray MF, Mahajan A, Nielsen JB (2018). Genetic inactivation of ANGPTL4 improves glucose homeostasis and is associated with reduced risk of diabetes. Nat Commun.

[19] Wang Y, Liu LM, Wei L, Ye WW, Meng XY, Chen F, Xiao Q, Chen JY, Zhou Y (2016). Angiopoietin-like protein 4 improves glucose tolerance and insulin resistance but induces liver steatosis in high-fat-diet mice. Mol Med Rep.

[20] Aryal B, Singh AK, Zhang X, Varela L, Rotllan N, Goedeke L, Chaube B, Camporez JP, Vatner DF, Horvath TL (2018). Absence of ANGPTL4 in adipose tissue improves glucose tolerance and attenuates atherogenesis. JCI Insight.

[21] Bouleti C, Monnot C, Germain S (2018). ANGPTL4, a multifaceted protein at the cross-talk between metabolism and cardiovascular disorders. Int J Cardiol.

[22] Mandard S, Zandbergen F, van Straten E, Wahli W, Kuipers F, Müller M, Kersten S (2006). The fasting-induced adipose factor/angiopoietin-like protein 4 is physically associated with lipoproteins and governs plasma lipid levels and adiposity. J Biol Chem.

[23] Robciuc MR, Naukkarinen J, Ortega-Alonso A, Tyynismaa H, Raivio T, Rissanen A, Kaprio J, Ehnholm C, Jauhiainen M, Pietiläinen KH (2011). Serum angiopoietin-like 4 protein levels and expression in adipose tissue are inversely correlated with obesity in monozygotic twins. J Lipid Res.

[24] Robciuc MR, Tahvanainen E, Jauhiainen M, Ehnholm C (2010). Quantitation of serum angiopoietin-like proteins 3 and 4 in a Finnish population sample. J Lipid Res.

[25] Janssen AWF, Katiraei S, Bartosinska B, Eberhard D, van Willems Dijk K, Kersten S (2018). Loss of angiopoietin-like 4 (ANGPTL4) in mice with diet-induced obesity uncouples visceral obesity from glucose intolerance partly via the gut microbiota. Diabetologia.

[26] Koliwad SK, Kuo T, Shipp LE, Gray NE, Backhed F, So AY, Farese RV, Wang JC (2009). Angiopoietin-like 4 (ANGPTL4, fasting-induced adipose factor) is a direct glucocorticoid receptor target and participates in glucocorticoid-regulated triglyceride metabolism. J Biol Chem.

[27] Dijk W, Heine M, Vergnes L, Boon MR, Schaart G, Hesselink MK, Reue K, van Marken Lichtenbelt WD, Olivecrona G, Rensen PC (2015). ANGPTL4 mediates shuttling of lipid fuel to brown adipose tissue during sustained cold exposure. Elife.

[28] Mattijssen F, Alex S, Swarts HJ, Groen AK, van Schothorst EM, Kersten S (2014). Angptl4 serves as an endogenous inhibitor of intestinal lipid digestion. Mol Metab.

[29] Ruppert PMM, Michielsen C, Hazebroek EJ, Pirayesh A, Olivecrona G, Afman LA, Kersten S (2020). Fasting induces ANGPTL4 and reduces LPL activity in human adipose tissue. Mol Metab.

[30] Jonker JT, Smit JW, Hammer S, Snel M, van der Meer RW, Lamb HJ, Mattijssen F, Mudde K, Jazet IM, Dekkers OM (2013). Dietary modulation of plasma angiopoietin-like protein 4 concentrations in healthy volunteers and in patients with type 2 diabetes. Am J Clin Nutr.

[31] Staiger H, Haas C, Machann J, Werner R, Weisser M, Schick F, Machicao F, Stefan N, Fritsche A, Häring HU (2009). Muscle-derived angiopoietin-like protein 4 is induced by fatty acids via peroxisome proliferator-activated receptor (PPAR)-delta and is of metabolic relevance in humans. Diabetes.

[32] Ortega-Senovilla H, van Poppel MNM, Desoye G, Herrera E (2018). Angiopoietin-like protein 4 (ANGPTL4) is related to gestational weight gain in pregnant women with obesity. Sci Rep.

[33] La Paglia L, Listì A, Caruso S, Amodeo V, Passiglia F, Bazan V, Fanale D (2017). Potential role of ANGPTL4 in the Cross Talk Between metabolism and cancer through PPAR signaling pathway. PPAR Res.

[34] Frayling TM, Timpson NJ, Weedon MN, Zeggini E, Freathy RM, Lindgren CM, Perry JR, Elliott KS, Lango H, Rayner NW (2007). A common variant in the FTO gene is associated with body mass index and predisposes to childhood and adult obesity. Science.

[35] Aryal B, Price NL, Suarez Y, Fernández-Hernando C (2019). ANGPTL4 in metabolic and cardiovascular disease. Trends Mol Med.

[36] Abid K, Trimeche T, Mili D, Msolli MA, Trabelsi I, Nouira S, Kenani A (2016). ANGPTL4 variants E40K and T266M are associated with lower fasting triglyceride levels and predicts cardiovascular disease risk in type 2 diabetic Tunisian population. Lipids Health Dis.

[37] Bailetti D, Bertoccini L, Mancina RM, Barchetta I, Capoccia D, Cossu E, Pujia A, Lenzi A, Leonetti F, Cavallo MG (2019). ANGPTL4 gene E40K variation protects against obesity-associated dyslipidemia in participants with obesity. Obes Sci Pract.

[38] McQueen AE, Kanamaluru D, Yan K, Gray NE, Wu L, Li ML, Chang A, Hasan A, Stifler D, Koliwad SK, Wang JC (2017). The C-terminal fibrinogen-like domain of angiopoietin-like 4 stimulates adipose tissue lipolysis and promotes energy expenditure. J Biol Chem.

[39] Oteng AB, Ruppert PMM, Boutens L, Dijk W, van Dierendonck X, Olivecrona G, Stienstra R, Kersten S (2019). Characterization of ANGPTL4 function in macrophages and adipocytes using Angptl4-knockout and Angptl4-hypomorphic mice. J Lipid Res.

[40] Gur-Cohen S, Yang H, Baksh SC, Miao Y, Levorse J, Kataru RP, Liu X, de la Cruz-Racelis J, Mehrara BJ, Fuchs E (2019). Stem cell-driven lymphatic remodeling coordinates tissue regeneration. Science.

[41] Kim HK, Youn BS, Shin MS, Namkoong C, Park KH, Baik JH, Kim JB, Park JY, Lee KU, Kim YB, Kim MS (2010). Hypothalamic Angptl4/Fiaf is a novel regulator of food intake and body weight. Diabetes.

[42] Galaup A, Gomez E, Souktani R, Durand M, Cazes A, Monnot C, Teillon J, Le Jan S, Bouleti C, Briois G (2012). Protection against myocardial infarction and no-reflow through preservation of vascular integrity by angiopoietin-like 4. Circulation.

[43] Aryal B, Rotllan N, Araldi E, Ramírez CM, He S, Chousterman BG, Fenn AM, Wanschel A, Madrigal-Matute J, Warrier N (2016). ANGPTL4 deficiency in haematopoietic cells promotes monocyte expansion and atherosclerosis progression. Nat Commun.

[44] Robciuc MR, Skrobuk P, Anisimov A, Olkkonen VM, Alitalo K, Eckel RH, Koistinen HA, Jauhiainen M, Ehnholm C (2012). Angiopoietin-like 4 mediates PPAR delta effect on lipoprotein lipase-dependent fatty acid uptake but not on beta-oxidation in myotubes. PLoS ONE.

